# A Built-In Strategy for Containment of Transgenic Plants: Creation of Selectively Terminable Transgenic Rice

**DOI:** 10.1371/journal.pone.0001818

**Published:** 2008-03-19

**Authors:** Chaoyang Lin, Jun Fang, Xiaoli Xu, Te Zhao, Jiaan Cheng, Juming Tu, Gongyin Ye, Zhicheng Shen

**Affiliations:** 1 Institute of Insect Sciences and State Key Laboratory of Rice Biology, Zhejiang University, Hangzhou, China; 2 Institute of Crop Sciences, Zhejiang University, Hangzhou, China; Massachusetts General Hospital, United States of America

## Abstract

Plant transgenic technology has been widely utilized for engineering crops for trait improvements and for production of high value proteins such as pharmaceuticals. However, the unintended spreading of commercial transgenic crops by pollination and seed dispersal is a major concern for environmental and food safety. Simple and reliable containment strategies for transgenes are highly desirable. Here we report a novel method for creating selectively terminable transgenic rice. In this method, the gene(s) of interest is tagged with a RNA interference cassette, which specifically suppresses the expression of the bentazon detoxification enzyme *CYP81A6* and thus renders transgenic rice to be sensitive to bentazon, a herbicide used for rice weed control. We generated transgenic rice plants by this method using a new glyphosate resistant 5-enolpyruvylshikimate-3-phosphate synthase (EPSPS) gene from *Pesudomonas putida* as the gene of interest, and demonstrated that these transgenic rice plants were highly sensitive to bentazon but tolerant to glyphosate, which is exactly the opposite of conventional rice. Field trial of these transgenic rice plants further confirmed that they can be selectively killed at 100% by one spray of bentazon at a regular dose used for conventional rice weed control. Furthermore, we found that the terminable transgenic rice created in this study shows no difference in growth, development and yield compared to its non-transgenic control. Therefore, this method of creating transgenic rice constitutes a novel strategy of transgene containment, which appears simple, reliable and inexpensive for implementation.

## Introduction

Transgenic crop improvement and modification is one of the core biotechnologies of modern agriculture [1]–[2]. Although extra measures have been taken in handling transgenic plants in field trials and commercial plantings to contain them, quite a few incidents of escapes have been reported in our very short history of transgenic agriculture practice [3]. Some specialized transgenic plants, such as pharmaceutical producing crops, could be potentially harmful if consumed unintentionally through contaminated food supply. For instance, ProdiGene Inc. was fined by the US government for not taking proper steps to prevent its pharmaceutical producing transgenic corn from entering the food supply [4].

In addition to physical containment measures, several genetic strategies, including maternal inheritance, male sterility and seed sterility (also called “terminator” technology), have been proposed for containing transgenes [5]–[6]. The chloroplast transformation as a maternal inheritance strategy has been successfully developed for various crops, including cotton, carrots and rice [7]–[10]. Although pollen was found to be able to transmit transgenes in chloroplast genomes at a very low rate in tobacco [11], chloroplast transformation is still considered as a sound containment strategy for pollen spreading [12]. The male sterility strategy was the only molecular technology that has been utilized in a commercial transgenic crop–rapeseed (Plant Genetic Systems, Ghent, Belgium). In this system, the pollen formation was inhibited by the expression of the ribonuclease barnase [13]. However, both maternal inheritance and male sterility strategy could only block pollen spreading but not seed dispersal by human errors, animal activity or other accidents. The “terminator” (US patent 5,723 765) and its alternative technologies [14]–[15] were designed to produce sterilized seeds, which germinate only if they are intentionally activated. However, the “terminator” technologies have not yet been demonstrated in field for major grain crops such as rice and corn. Thus, novel built-in containment strategies with high level of simplicity and reliability are still highly desirable for grain crops.

Containment of transgenic rice is an urgent issue. Transgenic rice not only could contaminate non-transgenic conventional rice, but also disperse to nearby weedy rice and other sexually compatible *Oryza* species, which co-occur with cultivated rice in field, through pollen-mediated gene flow. Although rice is primarily self-pollinating, the outcross between cultivated rice plants or between weedy rice and cultivated rice happens commonly [16]–[17]. Thus, the improved traits by transgenic modifications, such as greater resistance to biotic and abiotic stresses and greater tolerance to herbicides, could potentially pass to the weedy rice and its sexually compatible *Oryza* species to make them super weeds.

Bentazon is a benzothiadiazole herbicide which has been used for weed control of several major crops, such as rice, corn, wheat and soybean for over a decade. These crops are naturally resistant to the herbicide most likely because they express cytochrome P450 enzymes capable of detoxifying it [18]–[23]. Recently, the cytochrome P450 enzyme responsible for the detoxification of bentazon in rice was further identified as *CYP81A6* [19]. Thus we could generate bentazon sensitive rice plants by suppressing the expression of this detoxification gene thorough antisense RNA or RNA interference. Here we report the creation of the bentazon sensitive transgenic rice with high glyphosate tolerance. We demonstrated that such transgenic rice could be selectively eliminated by bentazon.

## Results

### Construction of a novel T-DNA plasmid for creation of terminable transgenic rice

**Figure 1:**
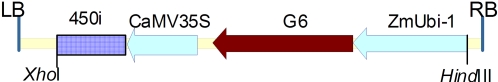
Diagram of the T-DNA fragment of the binary plasmid pG6-450i for *Agrobacterium* transformation. ZmUbi-1, *Zea mays* polyubiquitin-1 promoter; CaMV35S, cauliflower mosaic virus 35S promoter; *G6*, the 5-enolpyruvylshikimate-3-phosphate synthase isolated from *Pseudomonas putida* fused with chloroplast transit peptide at the N-terminus (gb: EU169459); R450i, the inverted repeat sequence of the 207bp fragment of *CYP81A6*; LB and RB, left and right border of the T-DNA.

We built a binary T-DNA transformation plasmid pG6-450i based on pCAMBIA1300. The T-DNA of pG6-450i consists of two parts, the gene of interest and the RNA interference cassette (Fig. 1). The gene of interest in this T-DNA is a glyphosate tolerance gene *G6* (gb: EU169459), which is a synthetic 5-enolpyruvylshikimate-3-phosphate synthase (EPSPS) gene from *Pseudomonas putida* fused with a chloroplast transit peptide at its N-terminus. The polyubiqitin-1 promoter of *Zea mays* [24] is used to drive the expression of this gene. *G6* serves as the transformation selection marker as well as the gene of interest for conferring glyphosate tolerance to facilitate weed control of the transgenic rice. The RNA interference cassette consists of the cauliflower mosaic virus 35S promoter (CaMV35S) and an inverted repeat sequence of 207bp of the rice cytochrome P450 gene *CYP81A6*. This RNA interference cassette was constructed in tandem to the *G6* gene inside the T-DNA (Fig. 1), so that they will be closely linked in transgenic plants.

### Rice transformation

The T-DNA plasmid pG6-450i was used to transform a local rice cultivar “Xiushui 110” (*Oryza sativa* L. *ssp. japonica*) by *Agobacterium*-mediated transformation method [25] using glyphosate as the selection agent. Total of over 40 independent transgenic events were obtained in our transformation. We noticed no significant difference in transformation efficiency compared to other transformation experiments using the same *G6* gene as the selection maker but without the RNA interference cassette, suggesting that this RNA interference cassette likely does not affect the transformation.

**Figure 2:**
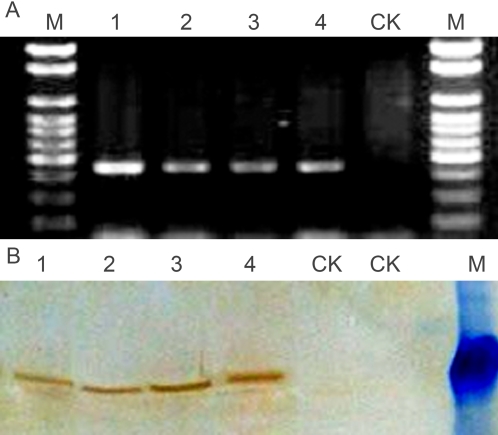
PCR and western analysis of transgenic T_0_ plants. The fragment of 440bp from the T-DNA insert was amplified from genomic DNA isolated from different events of transgenic rice (A). The G6 expressed in transgenic rice plants were detected by its antiserum from rabbits (B). Lane 1 to 4, transgenic event R450-2, R450-5, R450-6 and R450-7, respectively; CK, non-transformed rice as the negative control; M, 100bp ladder (A) or 48 kDa pre-stained protein size maker (B).

The insertion of the transgene in the selected transgenic events, R450-2, R450-5, R450-6 and R450-7, was further confirmed by PCR and immunoblot analysis (Fig. 2). PCR analysis with primers specific to the inserted DNA generated a product with the expected size of about 440bp (Figure 2A), while immunoblot analysis using the G6 (EPSPS) antiserum showed a specific band of about 45 kDa, which is the expected size of the G6 protein (Fig. 2B). The non-transgenic control plants were negative in both PCR and immunoblot analysis.

### Analysis of the sensitivity of the T_0_ transgenic rice plants against bentazon and glyphosate

The T_0_ plants from eight transgenic events were selected for determining their sensitivity to bentazon along with the non-transgenic plants. Primary screening spray with bentazon at 4000 mg/L indicated that 7 of the 8 events tested were sensitive to bentazon. To determine the minimum dose required for terminating the transgenic rice, the transgenic plans of the four events, R450-2, R450-5, R450-6 and R450-7, were then further tested with spray of bentazon at 500, 1000 and 2000 mg/L, respectively. We found that one spray of bentazon at 1000 mg/L or higher killed 100% transgenic plants of all the four events in 7 days, while a spray of 500 mg/L did not kill any of the transgenic plants in the greenhouse. Thus the minimum dose required for the termination of these transgenic plants is about 1000 mg/L, which is within the regular dose range used for normal rice weed control.

**Figure 3:**
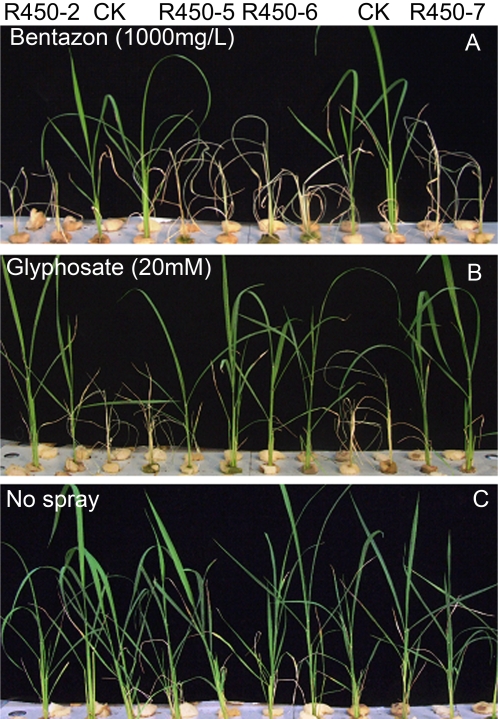
Sensitivity test of transgenic rice plants to bentazon or glyphosate. The T_0_ transgenic rice plants along with non-transgenic ones were cultured in solution in greenhouse and sprayed with 1000 mg/L bentazon (A) or 20 mM glyphosate (B). The plants in panel C were not sprayed. The pictures were taken 7 days after the spray. Independent transgenic events (R450-2, R450-5, R450-6, and R450-7) along with untransformed rice (CK) were tested. The plants in each panel were in the same order.

To further characterize their sensitivity to both bentazon and glyphosate, the four transgenic events, R450-2, R450-5, R450-6, and R450-7, were sprayed with bentazon at 1000 mg/L and glyphosate at 20 mM in greenhouse, respectively. We found that the spray of bentazon of 1000 mg/L killed all the 6 transgenic plants of each events tested but not any of the conventional rice in 7 days (Fig. 3A). In contrast, the 20 mM glyphosate spray killed all the conventional rice plants but not any of the transgenic rice plants in 7 days (Fig. 3B). The transgenic plants that were not sprayed with either bentazon or glyphosate appeared to be healthy and grew normally (Fig. 3C).

Therefore, the transgenic rice plants we generated were highly sensitive to bentazon but tolerant to glyphosate, which is exactly the opposite of conventional rice plants. This striking difference in response to the two herbicides between the transgenic and the non-transgenic rice makes the selection or termination of the transgenic rice plants to be extremely convenient and effective.

### The RNAi suppression of the bentazon detoxification gene *CYP81A6*

**Figure 4:**
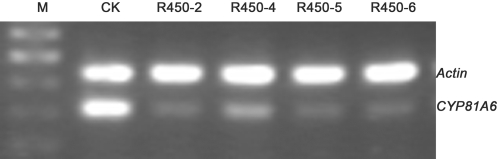
RT-PCR analysis of *CYP81A6* mRNA abundance in transgenic and non-transgenic rice. The rice *Actin* gene was used as the control. M, DNA size maker; CK, non-transgenic rice; R450-2, R450-5, R450-6 and R450-7 are independent transgenic events sensitive to bentazon.

RT-PCR was used to estimate the relative abundance of the *CYP81A6* mRNA in transgenic rice and non-transgenic rice. The total RNAs were isolated from the non-transgenic control plants and the transgenic plants of each event of R450-2, R450-5, R450-6, and R450-7, respectively. The total RNAs were used as the templates for the one-step RT-PCR analysis of the rice *CYP81A6* gene and *Actin* gene. We found that the amount of the 310bp *CYP81A6* RT-PCR product was greatly reduced from the transgenic plants compared to that from the non-transgenic control plants, while the amount of the 440bp *Actin* RT-PCR product was roughly equal among the transgenic and non-transgenic plants (Fig. 4). This result suggested that the *CYP81A6* mRNA in these transgenic plants was significantly suppressed. This is in agreement with our assumption that the RNA interference cassette introduced in tandem with the gene of interest in these transgenic rice plants is responsible for their sensitivity to bentazon.

### Field trial for the terminable transgenic rice

**Figure 5:**
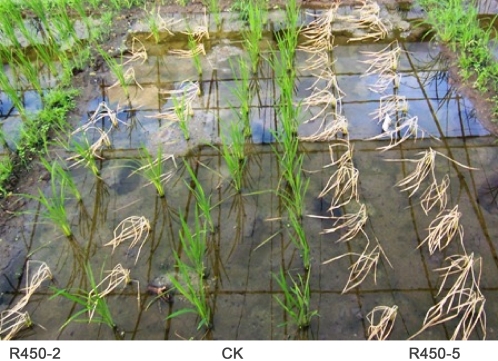
Field trial for selective termination of the transgenic rice. The T_1_ transgenic plants and the conventional control rice plants were sprayed with Bentazon at 1500 mg/L. The picture was taken 7 days after the spray. CK, conventional rice; R450-2 and R450-5 are T_1_ transgenic rice plants of the event R450-2 and R450-5, respectively. The surviving plants in the R450-2 rows were segregates not carrying the transgene.

The T_1_ plants of events R450-2 and R450-5 were further tested for their sensitivity to bentazon in a field trial. The T_1_ seedlings of the events R450-2 and R450-5 along with the non-transgenic conventional rice seedlings of the same cultivar were replanted individually in the trial field. All of the individual F1 plants were analyzed by PCR to determine if they were transgenic plants or segregates without the transgene. Thirteen of the total 23 individually replanted plants of the event R450-2 are transgenic, while all of the plants of the event R450-5 are transgenic, as determined by PCR analysis. Fifteen days after replanting, bentazon of 1500mg/L was sprayed at the rate of 100 mL/m^2^. All of the transgenic plants of both events R450-2 and R450-5 died within 6 days after spray, while all the conventional rice plants and the negative segregates without the transgene survived as expected (Fig. 5). The event R450-5 may have multiple copies of insert in its genome, which may explain the lack of negative segregates in its T1 population.

Furthermore, we did not observed any yield penalty or other abberations of phenotype over the transgenic plants. We measured the plant height, number of panicles per plant, average panicle length, number of grains per panicles and weight of per 1000 grains of the F1 transgenic rice plants of both event R450-2 and R450-5 (Table 1). We found no statistical difference (at p<0.01) between the transgenic and non-transgenic plants in all these parameters as assessed by Duncan's multiple–range test using the DPS statistical software, suggesting that the suppression of *CYP81A6* expression does not have statistically significant side effects on the performance of the transgenic rice in field.

**Table 1:** Comparison of agronomic traits between the terminable transgenic rice and its non-transgenic control in field trial.

	Plant height (cm)	Panicles per plant	Panicle length (cm)	Grains per panicle	Weight per 1000 grains (g)
Control	65.43	10.35	18.25	56.08	27.03
R450-2	64.38	10.18	17.32	59.98	27.07
R450-5	66.00	10.86	17.60	54.21	26.99

Control plants are non-transgenic rice of the same cultivar as the transgenic plants. R450-2 and R450-5 are two independent transgenic events.

## Discussion

Once an event of transgenic rice is released for commercial planting, it is hard to ensure the total containment. Thus, it is important to develop technology to selectively terminate the escapes to ensure the decontamination of the concerned fields. Normally it is difficult even to detect let alone to selectively terminate the transgenic plants from the non-transgenic ones in large area of crops. However, the strategy we report here will make the detection and selective termination of the transgenic rice plants quite convenient. In this strategy, the transgenic rice could be detected and terminated selectively by a registered agrichemical.

This unique technology is especially useful for creating transgenic rice plants as bioreactors for molecular farming, an emerging technology for production of pharmaceutical and industrial proteins. Currently many pharmaceutical proteins produced from plants are under clinical trials [26]. These otherwise high value proteins could be harmful if consumed unintentionally though the contamination from food or feed supply. This novel containment technology could in essence serves as an insurance policy of no contamination for food or feed supply. Therefore, we suggest that all transgenic rice for molecular farming should be generated with a controllable maker such as the one described in this report.

Moreover, this technology may also be used for creating transgenic rice with genes that are currently regarded as safe. The safety regarding a gene used in a transgenic crop may be conditional and subject to change with our better understanding over time. A safe gene now could be considered as undesirable in the future. Transgenic plants created by this strategy could make any recall of a released transgene much easier if ever need.

This novel strategy appears simple, reliable and inexpensive for implementation. To create terminable transgenic rice, the T-DNA binary plasmid pG6-450i reported in this paper could be used as a basic backbone for inserting any genes of interest. In addition to the RNA interference cassette for conferring sensitivity to bentazon, this T-DNA plasmid contains an EPSPS gene for selection in transformation as well as for conferring glyphosate tolerance. Moreover, this strategy does not require protein encoding genes to be introduced into transgenic plants to carry out the strategy as the “terminator” technology normally does.

This strategy is also highly reliable. The gap of the killing dose of bentazon between the transgenic and non-transgenic plants is quite wide. The minimum dose for transgenic rice plants generated in this report is about 1000 mg/L, while that for the non-transgenic rice could be as high as 5800 mg/L [22]. This wide gap will make the termination of transgenic rice plants quite feasible and flexible. Our field trials demonstrated that all the transgenic rice plants were killed efficiently by one spray of bentazon at regular weed control dose. Thus, the reliability of this strategy is as high as that of bentazon for weed control.

Finally, because bentazon is an herbicide that has been registered for weed control in rice, it is ready for use. There will be almost no extra cost incurred for decontaminating transgenic rice if bentazon is incorporated into the practice of weed control for conventional rice production. Importantly, this method may be applicable to other grain crops, especially corn. Currently we are working on field trials for the transgenic terminable corn.

While this study clearly demonstrated that this transgene containment strategy is feasible, more detailed studies are required to calibrate the bentazon dose required for terminating transgenic rice plants of different genetic background, growth conditions and growth stages. Also, the increased utilization of the bentazon by the deployment of this technology may be a concern for environmental safety and weed resistance development.

## Materials and Methods

### T-DNA plasmid construction

The 207bp fragment of *CYP81A6* DNA was obtained by PCR from the rice genomic DNA isolated from cultivar “XiuShui 110” using primer 450F ( CTCGAGCAGTGCACCAGAGTCACAGAAACACATCACAC (an *Xho*I site was attached and underlined), and 450R (5′AGATCTGCT TCTTGACGAGGTGGAGGTGT, a *Bgl*II site was attached and underlined). This fragment represents the very 5′ end of the *CYP81A6* cDNA from 1 to 207bp. Another 327bp fragment of *CYP81A6* cDNA was obtained by PCR from the same rice genomic DNA using the primer 450F, and the primer 450R2 (5′ AGA TCTCGGTGAAGCACTCCCTGGCGCAC, a *Bgl*II site was attached and underlined). This fragment represents the very 5′ end of the cDNA from 1 to 327bp. Both PCR products were cloned into the pMD-T vector (Shanghai Sangon, China), confirmed by sequencing and then released from the T-vectors by digestion with *Xho*I and *Bgl*II. These two fragments and the T-DNA plasmid pCAMBIA1300 predigested with *Xho*I and dephosphorated, were ligated. The resulted plasmid construct, which contains a 207bp inverted repeat sequence of *CYP86A6* for RNA interference, was named as p1300-450i.

The full length of maize optimized synthetic glyphosate resistant EPSPS gene *G6*, including its 5′ end DNA fragment encoding the chloroplast transit peptide from the acetohydroxyacid synthase of *Zea mays* and its 3′ end terminator fragment from the maize phosphoenolpyruvate carboxylase, was synthesized by Shanghai Sangon Limited, China (gb: EU169459). *G6* was isolated from *Pesudomonas putid* in our laboratory recently. The restriction sites of *Bam*HI and *Eco*RI were added to its 5′ and 3′ end of the gene, respectively, to facilitate the cloning. The *Z. mays* polyubiquitin-1 promoter [25], ZmUbi-1, was obtained by PCR using primer ZmUbiF (5′ GCGAAGCTTGCATGCCTACAGTGC AGCGTGACCCGGTCGTGC, a *Hind*III site was attached and underlined) and ZmUbiR (5′ GTGGGATCCTCTAGAGTCGACCTGCAGAAGTAACACCAAACAACAG, a *Bam*HI site was attached and underlined). Advantage GC cDNA PCR kit (TaKaRa) was used to obtain this promoter by PCR from maize genomic DNA. This PCR amplified ZmUbi-1 promoter was digested with *Bam*HI and *Hind*III, and then ligated in a 3-way to the synthetic *G6* gene predigested with *Eco*RI and *Bam*HI, and the plasmid p1300-450i predigested with *Hind*III and *Eco*RI. The resulted T-DNA construct was named as pG6-450i and was used for *Agribacterium* mediated rice transformation.

### Rice Transformation

T-DNA plasmid transformation vector pG6-450i was transformed into *Agrobacterium tumefaciens* (LAB4404) by electroporation. A local rice cultivar “Xiushui-110” (*Oryza sativa* L. *ssp*. *japonica*) was transformed using an *Agrobacterium*-mediated transformation procedure described previously [25]. Glyphosate of 2–3 mM final concentration was used as the selection agent for tissue culture media. The rooting media was also added with 0.1 mM glyphosate for further selection.

### Culture of transgenic rice

T_0_ transgenic rice plants of independently transformed events were cultured in a greenhouse in solution prepared according to Yoshida et al. (1976) at about 18–25C° with 12–14 h light [27]. Herbicide spray tests were carried out when the plants reached to 7–10 cm in height. To obtain the T_0_ plant seeds, the transgenic rice plants from the solution culture were replanted to soil containers in greenhouse. For field trials of T_1_ plants, the T_0_ seeds were first geminated and grew on soil seedbed for 3 weeks, and then the seedlings were replanted into the test field.

### Spray of herbicides

The rice plants were all sprayed with handhold sprayer at the rate of 100 mL/m^2^. Bentazon (48% solution) was obtained from Jiangsu Luli Limited (Jiansu, China). In primary screening, bentazon of 4000 mg/L was used for spraying. In sensitivity assay, bentazon with final concentrations of 500, 1000 and 2000 mg/L were sprayed evenly. Glyphosate was obtained from Albaugh Inc. (Iowa, USA). It was diluted to 20 mM and then added with Tween-20 to the final concentration of 0.01% for spray. For field trials, bentazon of 1500 mg/L were sprayed at the rate of 100 mL/m^2^ also, and the growth of the plants was monitored daily.

### PCR analysis

CTAB method [28] was used for the isolation of genomic DNA from rice leaves. The primers for PCR are 35S-R (5′ CTCGAAGCTTACGTTTTTAATGTAC TGAAT) and 450R (5′AGATCTGCTTCTTGACGAGGTGGAGGTGT), which could amplify a DNA fragment of 440bp, consisting of the CaMV35S terminator and the 207bp of the sequence of *CYP81A6*. The PCR products were analyzed by agarose gel electrophoresis.

### Western analysis

Standard western analysis method was carried out to detect the expression of G6 in transgenic rice plants. Leaf samples collected from transgenic plants as well as non-transgenic control plants were ground in liquid nitrogen and then suspended in SDS sample buffer. After boiled for 10 min, the soluble fractions of these samples were separated by SDS-PAGE and then blotted onto nitrocellulose membrane. The rabbit antiserum against G6 was used as the first antibody and the alkaline phosphatase-conjugated goat anti-rabbit IgG as the second antibody (Sigma).

### RT-PCR analysis

Five rice plants from each transgenic event and non-transgenic control plants of the same cultivar were sampled at 20 days after germination. The leaves collected from the 5 plants of the same event or the non-transgenic control were combined and its total RNA was extracted with the TRIzol reagent (Invitrogen). RT-PCR was performed using one-step RT-PCR kit (Fermentas). A cDNA fragment of *CYP81A6* was amplified by the primer R450A1 (5′ TCGCCGCCTGATCGACGCGGAGCGGC) and R450A2 (5′ TGCACTGGAGGTAGCCGAGGCGAGT). The corresponding genomic fragment of this cDNA contains an intron, thus we can rule out the possible contamination of genomic DNA in the PCR reaction. Meanwhile, the *Actin* cDNA fragment was amplified, as a control, in a separate reaction with the same RNA as template using primer Act1 (5′AGGGCTGTTTTCCCTAGTATCGTGG) and Act2 (5′GATGGCATGAGGAGGGGCAT). The PCR products of *CYP81A6* and *Actin* from the same event or control plants were then combined and analyzed by argrose gel electrophoresis.

### Field trials

Transgenic rice plants as well as the non-transgenic control plants were planted and tested in field from May 2006 to Oct. 2007 at the University farmer of Zhejiang University at Hangzhou, China. The biometrical data on plant height, number of panicles per plant, panicles length, number of grains per panicles and weight per 1000 grains were measured, recorded and the mean values were analyzed by Duncan's multiple–range test using the DPS statistical software (Refine Information Tech. Inc., Hangzhou, China).
